# Freezing our way to a cure?

**DOI:** 10.1016/j.igie.2024.04.013

**Published:** 2024-04-26

**Authors:** Linda S. Lee, Bill Busby, Vivek Kaul

**Affiliations:** 1 Brigham and Women's Hospital, Boston, Massachusetts, USA; 2 STERIS Corp, Mentor, Ohio, USA; 3 University of Rochester Medical Center, Rochester, New York, USA

## Editor’s introduction

In the mid 19th century, Dr James Arnott’s pioneering work laid the early foundations for the use of cryotherapy in medicine.^1^ Initially used for the treatment of neuralgia and migraine headaches, Arnott’s innovative approach expanded the application of cryotherapy to the treatment of various malignancies including breast cancer, uterine cancer, and skin cancer.

In the modern era, endoscopic cryotherapy (EC) represents a significant advancement in endoscopic practice for the management of a variety of benign and malignant conditions. In 2005, Johnston et al^2^ introduced EC as a novel technique for the treatment of dysplastic Barrett’s esophagus (BE), marking a pivotal moment in gastroenterologic innovation in this disease state. By delivering liquid nitrogen spray cryotherapy (LNSC) to the esophagus through a specialized catheter inserted through a standard gastroscope, they demonstrated its efficacy in targeting focal areas of esophageal dysplasia. The mechanism of action, which involves rapid cycles of cooling and thawing, induces cellular disruption, vascular injury, and apoptosis (while preserving the collagen matrix) and ultimately leads to eradication of dysplastic tissue.^3^

Presently, 2 distinct EC platforms are available. Liquid nitrogen–based spray cryotherapy (STERIS Corp, Mentor, Ohio, USA), uses a specialized catheter to deliver liquid nitrogen at –196°C to the targeted tissue by administering multiple freezing sprays per site, with each spray followed by a timed thaw interval. Because of the rapid expansion of nitrogen gas, a dedicated peroral decompression tube is used to vent the stomach and reduce the risk of gastric perforation. A relatively newer EC delivery system uses an innovative cryoballoon focal ablation system (Pentax Medical, Redwood City, Calif, USA). This system uses liquid nitrous oxide, which is converted to gas within a single-use, low-pressure, compliant balloon with freezing temperatures reaching up to –85°C to ablate the tissue.

Advancements in EC technology and research have enabled its widespread application in clinical practice in recent years, with compelling evidence supporting its efficacy across diverse GI pathologies. From addressing dysplastic BE, radiation proctitis, and GI bleeding to managing refractory benign esophageal strictures, and duodenal polyps and providing palliative care for esophageal cancer, EC has emerged as a versatile tool in the gastroenterologist’s armamentarium.^4^, ^5^, ^6^^,^^7^

In this Industry Perspective article, Bill Busby, Vice President and General Manager, Procedural GI for STERIS, provides valuable insights into the historical perspective of EC development, the current state of the technology, and what the future might hold in this realm. In his role, he is responsible for the overall management of the business unit, which includes the development, manufacture, and commercialization of products and technology for the procedural environment of endoscopy. Bill earned his BA in psychology from Wittenberg University and a JD from Capital University School of Law. Before assuming his current role as general manager in 2019, Bill held various roles in the business, ranging from human resources to sales and marketing, and has been with the business for 18 years.

—Linda S. Lee, *iGIE* Section Editor for Industry Perspectives; Vivek Kaul, Associate Editor, Insights

**Linda Lee: Thank you very much for making time with your incredibly busy schedule to discuss the evolution of cryoablation in GI. Would you discuss why there was interest in bringing cryoablation into GI and how it became a focus for your company at STERIS?**

**Figure 1:**
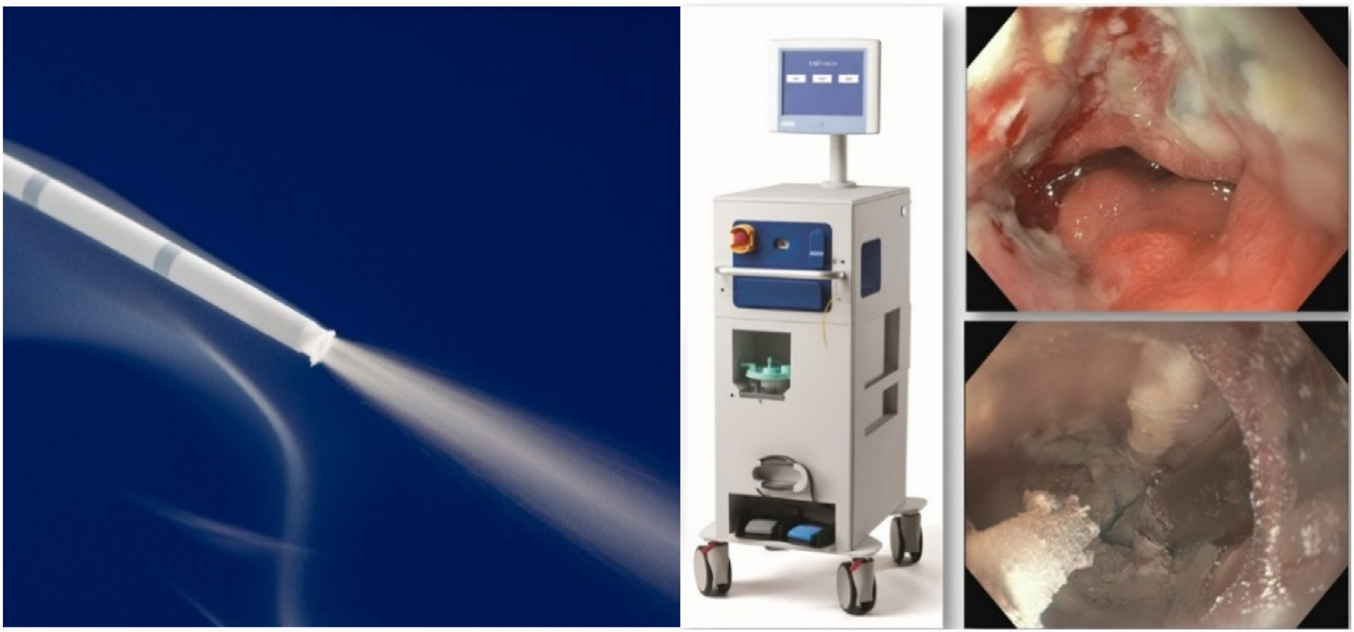
The truFreeze cryotherapy catheter, device, and endoscopic images.

**Bill Busby:** LNSC originated from the innovative work of Dr Mark Johnston, a Navy gastroenterologist, during the 1990s. Initially developed as an alternative to esophagectomy for BE with high-grade dysplasia, its scope expanded as further research and evidence emerged. Beyond its primary application, liquid nitrogen has been found to be effective in treating esophageal cancers across various stages, from early disease for curative intent to palliation in late-stage disease as an alternative to esophageal stents. Its versatility and efficacy in treating GI tract conditions aligns with our patient-centric approach in the GI space. The truFreeze cryotherapy platform (STERIS Corp, Mentor, Ohio) (Fig. 1) stands as a cornerstone therapeutic technology, offering a comprehensive solution for eligible patients. We believe adding it to our existing portfolio of high-quality products aligned with our organization’s mission to create a healthier and safer world using progressive technology.

**LL: Would you discuss the key steps in developing a cryoablation system that could be used in GI?**

**Figure 2:**
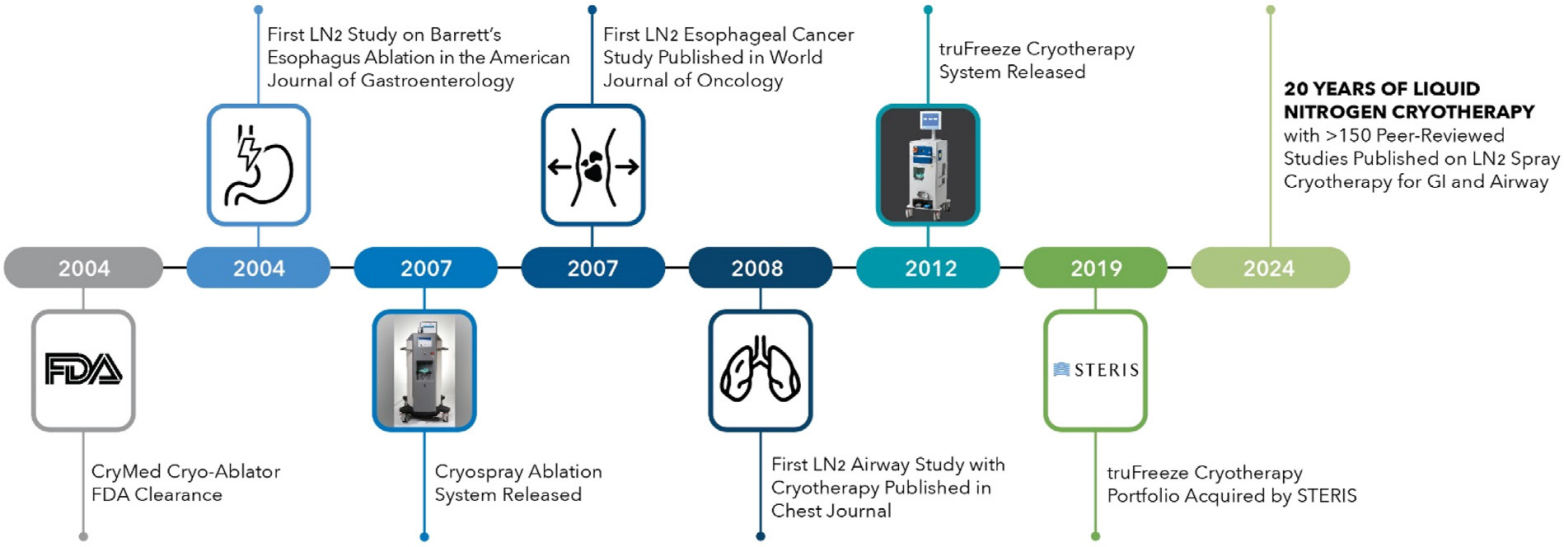
Timeline of key events in cryoablation for gastroenterology. *FDA*, U.S. Food and Drug Administration; *LN*_*2*_, liquid nitrogen.

**BB:** STERIS acquired truFreeze cryotherapy in December 2019 (Fig. 2). Much of the initial testing required for the novel concepts of using liquid nitrogen inside the body to treat disease was performed prior to our acquisition. Liquid nitrogen is the coldest cryogen available, at –196°C, and it also expands rapidly (7×) as it transitions from a liquid state to a gaseous form. From a development standpoint, these are the critical items to balance with regard to manufacturing and safety.

STERIS has been building upon the foundation technology. Our engineering team has deepened their knowledge of cryogenics and is focused on future advancements, such as improving the ease of delivery and accessing how this technology can be delivered to other target sites in the body. Our clinical research team is engaging with physicians on innovative approaches and applications for using LNSC in both the GI tract and in the respiratory system. The year 2024 now marks 20 years since the initial submission of this technology to the U.S. Food and Drug Administration. We continue to make advancements in improving the technology and expanding clinical research to support the use of this technology across a wide range of clinical applications.

**LL: What were the challenges you faced when your company acquired this technology, and how did STERIS overcome these?**

**Figure 3:**
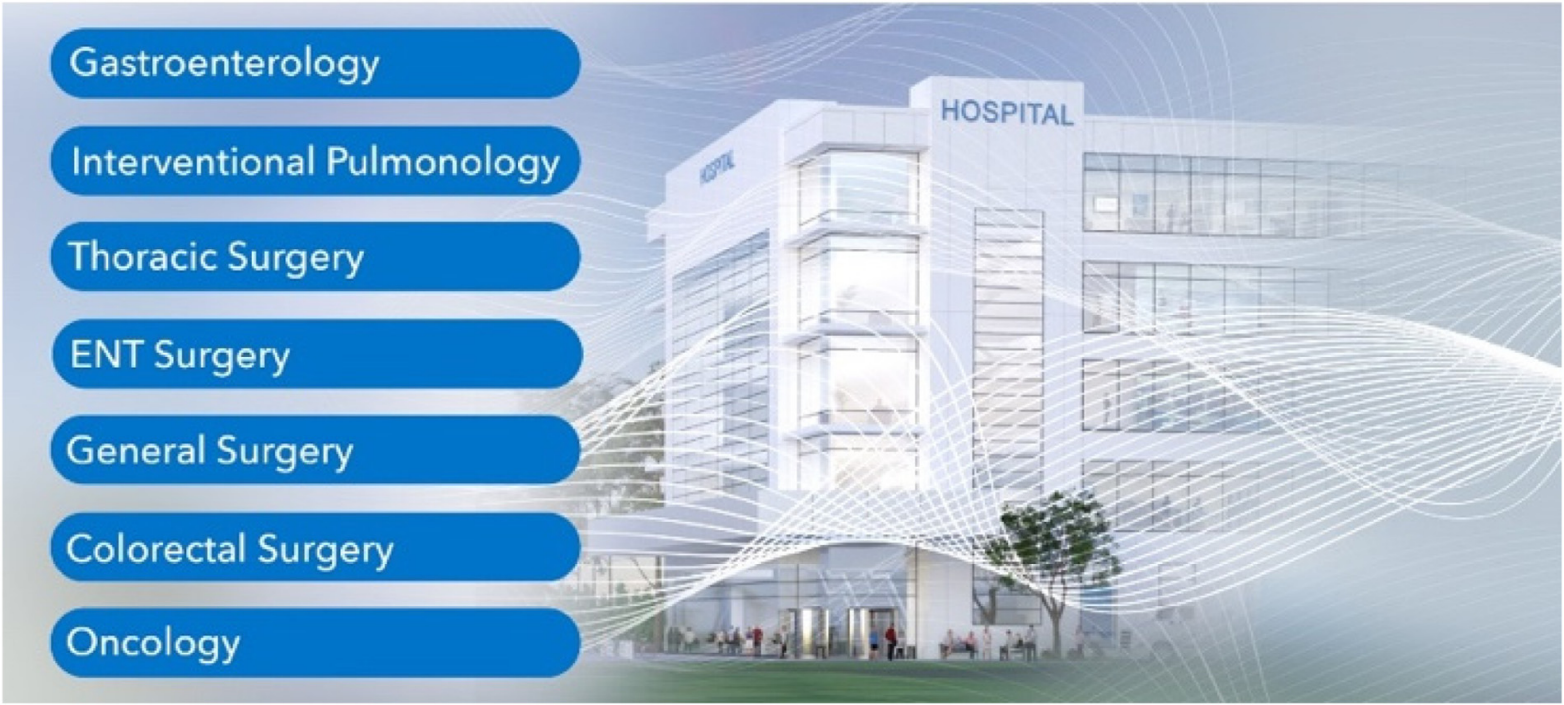
Application of liquid nitrogen spray cryotherapy in medicine. *ENT*, Ear, nose, and throat.
(2) We had to scale our organization to manage the rapid growth of this technology. Although the science behind the technology itself has not changed, there has been extensive new research in the esophageal cancer space, which propelled the adoption of LNSC in GI. For example, LNSC is now used in 9 of the top 10 cancer centers in the United States. We see similar growth with pulmonary by interventional pulmonologists and ear, nose, and throat surgeons with the ongoing research on CryoStasis for tracheal stenosis. We have received requests from physicians and patients in Europe, Asia, and South America to make this technology available, and it’s been a challenge to expand fast enough.
(3) Clinicians have shared that they would like to see more innovation with LNSC to enhance the technology, improving ease of use and providing access to treat more areas of the body. The current system is constrained by where we can actively or passively vent the nitrogen gas.

**BB:** With any acquisition, there is a learning curve associated with familiarizing ourselves with the technology and customer needs. We conducted a thorough review of the documentation to support the truFreeze system and catheters, and it was then integrated into our core regulatory framework. Experienced clinical and sales team members joined STERIS at acquisition, so we were able to support an uninterrupted transition for hospital systems and clinicians currently using LNSC. We went to medical facilities and worked alongside clinicians using LNSC in practice to better understand the benefits and limitations of the therapy. At the beginning, we learned that we faced 3 key challenges with truFreeze cryotherapy:

(1) truFreeze cryotherapy is used by many specialties across the hospital for a diverse array of applications in the airway and GI tract. We needed to quickly expand our field clinical staff to adequately support our customers in procedures. The pulmonary and surgical specialties were new to our organization, so we spent the first couple of years learning from clinicians how to better support LNSC cases in these areas. LNSC is clinically relevant to 7 medical specialties (Fig. 3).

**LL: What were the unique challenges in working with this product?**

**BB:** The use of liquid nitrogen in modern medicine dates back to the 1950s. Dermatologists and other medical disciplines have used it for everything from tissue preservation to treating melanoma and other cancers. Physicians have discovered that liquid nitrogen effectively ablates unwanted benign tissue and cancers, leaving minimal scarring. However, its ultracold nature presents a unique challenge: it boils at –196°C, transitioning rapidly to nitrogen gas when exposed to warmer temperatures. Although nitrogen gas constitutes 78% of the air we breathe, its conversion within the patient creates inherent gas management (ventilation) challenges. Multiple iterations of suction methods were developed to effectively evacuate the gas from the patient. Moreover, there is a limited selection of component materials capable of withstanding such extreme temperature fluctuations while remaining pliable and functional.

**LL: Would you discuss what your strategy has been in introducing this product globally?**

**BB:** Currently, truFreeze is available in the United States and is used by hundreds of health care facilities across the country. Our strategy is to focus on supporting these medical systems with the adoption of LNSC into the standard treatment of esophageal cancer patients across gastroenterology, oncology, and thoracic surgery. Our goal is to make cryotherapy available at every medical center treating patients with esophageal cancer.

More than 50,000 people in the United States are living with esophageal cancer, and the majority of these patients are diagnosed in advanced stages and have difficulty eating or swallowing.^8^^,^^9^ Through our research, it’s apparent there is an unmet need to offer these patients additional treatment options to improve outcomes and quality of life. Expanding the awareness of the available research supporting LNSC in the application for esophageal cancer is at the heart of what we do. Medical specialties can sometimes become siloed, so we are partnering with oncologists to broaden the education of LNSC as a tool for treating esophageal cancer in parallel with the standard-of-care systemic therapies.

We also recognize that patients outside of the United States can benefit from truFreeze cryotherapy. In fact, we are contacted by patients from all over the world about truFreeze. Many have seen the patient testimonials from the news or on our website and ask their physicians about LNSC. Today, we connect them with physicians in the United States for treatments with LNSC; however, we know this isn’t enough. We have a therapy that can help physicians treat patients, and we feel it’s our responsibility to get this tool in their hands. We have plans to make truFreeze spray cryotherapy more widely available starting by its introduction outside the United States this year.

**LL: Radiofrequency ablation (RFA) remains widely used to treat BE with dysplasia and other conditions including gastric antral vascular ectasia and radiation proctitis. How do you see cryoablation fitting into this landscape, and where do you see opportunities for the growth of cryoablation?**

**Figure 4:**
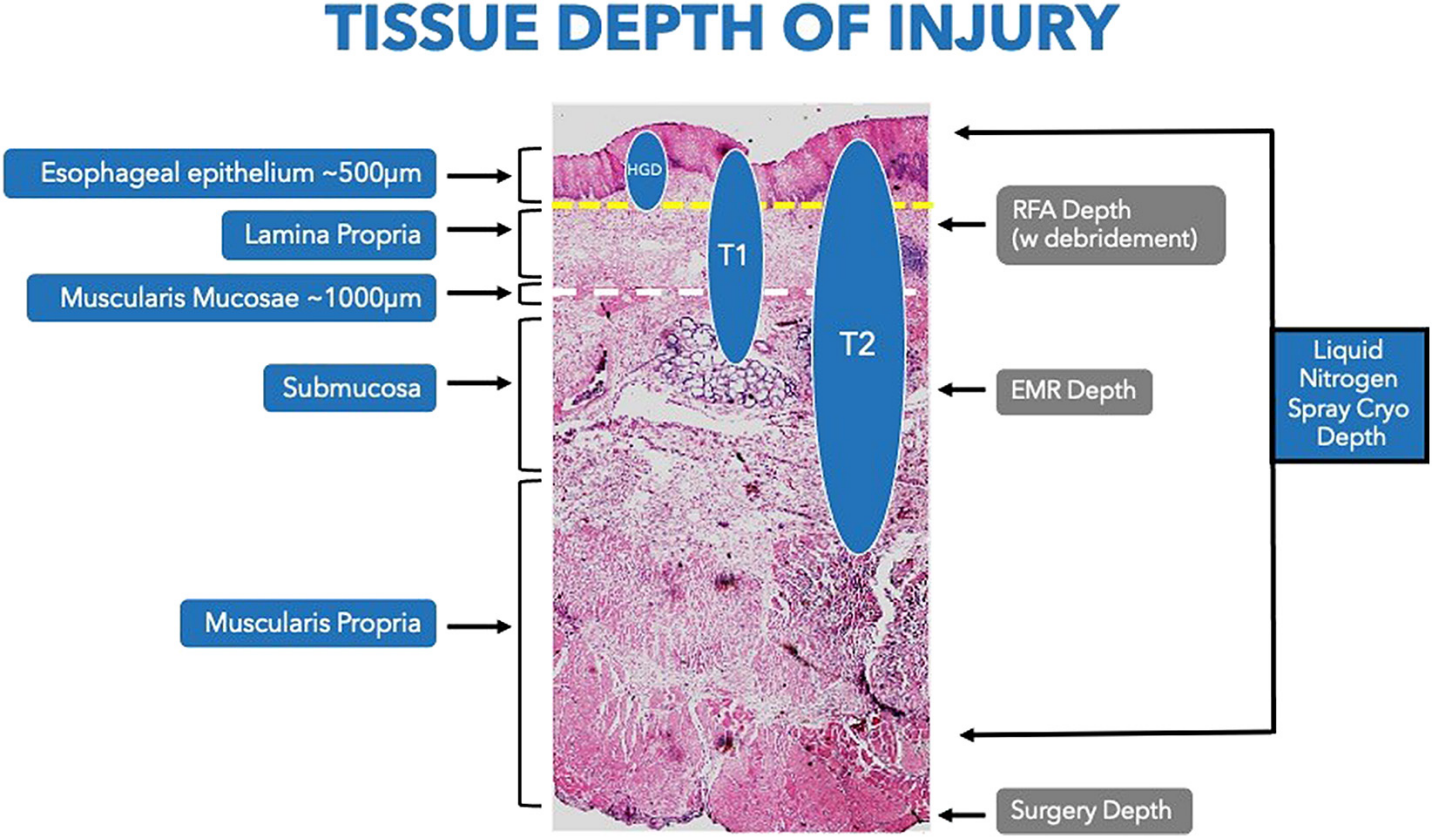
Depth of tissue injury with different endoscopic techniques. *HGD*, high-grade dysplasia; *T1*, cancer invading the submucosa; *T2*, cancer invading the muscularis; *RFA*, radiofrequency ablation.

**Figure 5:**
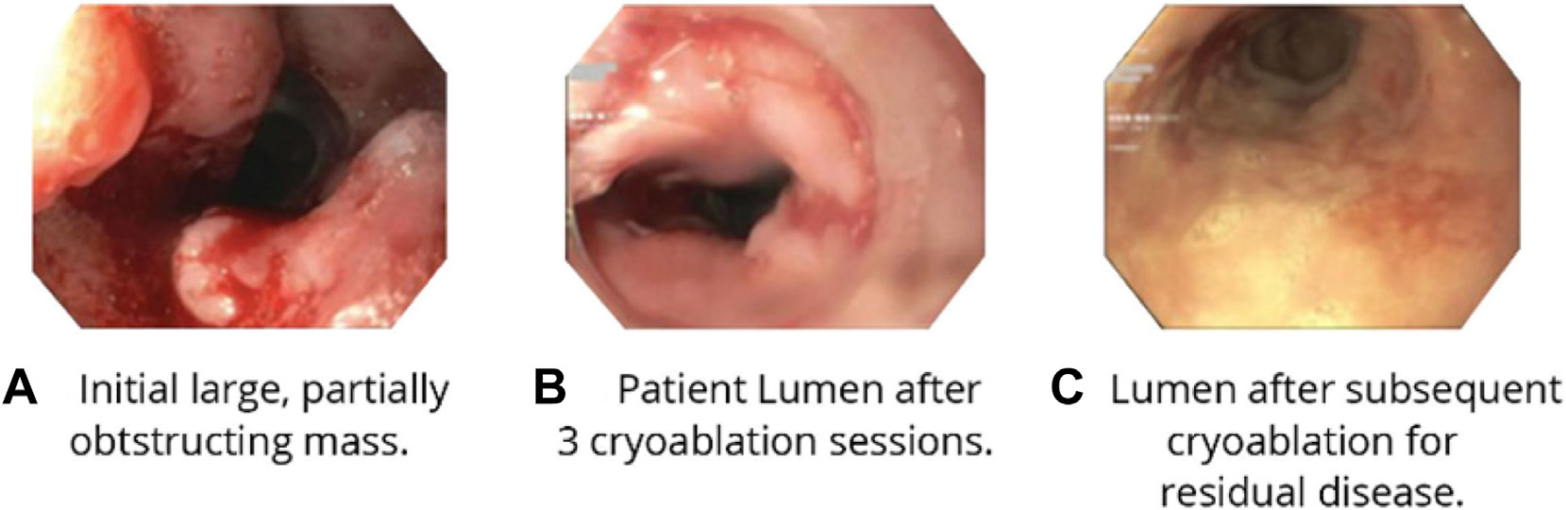
Endoscopic image of mass in squamous cell esophageal cancer.

**BB:** Although the initial concept of cryotherapy was developed for treating BE, its success has expanded to the treatment of esophageal cancer. Because of the extremely cold nature of liquid nitrogen and the flexibility in dosing, truFreeze cryotherapy is able to treat deeper relative to other ablation technologies (Fig. 4).^10^ In addition, LNSC is noncontact, which enables physicians to treat tumors of various sizes and shapes, such as squamous cell esophageal cancer, depicted in Figure 5.

Esophageal cancer patients have limited treatment options available to help manage symptoms like dysphagia. Treatments such as stents and radiation carry considerably higher rates of side effects, adverse events, and toxicity to patients.^11^^,^^12^ The research available on LNSC demonstrates its safety and good patient tolerance at any stage of esophageal cancer. Patients benefit from improved quality of life and dysphagia relief while also avoiding or delaying the need for a stent.^12^, ^13^, ^14^, ^15^ It is rewarding to hear the success stories and know patients are able to enjoy meals with their families in part because of this technology.

In addition to treating dysplastic BE and esophageal cancer, LNSC can be used as an ablation treatment for gastric cancer, gastric antral vascular ectasia, and radiation associated vascular ectasia, among other conditions. An emerging application where we’re supporting ongoing clinical research is refractory stricture management in the esophagus and in the airway. LNSC has been used for more than a decade to treat tracheal and bronchial stenoses. For example, several major U.S. health care centers use LNSC to treat lung transplant patients with postanastomotic strictures using a technique called CryoDilation. Interventional pulmonologists use LNSC followed by balloon dilation to expand the airway and reduce the frequency of dilations needed to maintain airway patency in patients. The same premise can be used for postanastomotic refractory strictures in patients who have undergone esophagectomies or are experiencing peptic or radiation strictures that will not resolve with balloon dilation alone. New data were presented on this application at Digestive Disease Week in May 2024.^16^

The incidence of BE is on the rise, and LNSC has been shown to be effective and well tolerated as a salvage treatment in patients who for whom therapy with RFA fails.^17^^,^^18^ Additionally, LNSC has the advantage of treating nodular mucosa, which has been proven to be challenging in RFA, which requires tissue contact.

In the most recently published prospective, multicenter (11 academic sites) registry on treating BE with LNSC, nearly 33% of patients had previously undergone RFA before LNSC. These patients achieved complete eradication of dysplasia rates of over 80%, demonstrating the efficacy of salvage LNSC in a group where treatment options are limited. Furthermore, this was the largest prospective dataset (n = 138 patients) evaluating LNSC for the treatment of BE. The data from this registry showed that after 3 years, the patients who were RFA naive had a complete eradication of dysplasia of 96% and complete eradication of intestinal metaplasia of 77%. These most recent data demonstrate that cryotherapy is a viable ablation treatment for both first-line therapy in BE and for refractory Barrett’s disease.^19^ Beyond the efficacy of treatment durability, LNSC also offers less postprocedural pain and a lower reported posttreatment stricture rate relative to other endoscopic ablation options.^20^^,^^21^

**LL: Some of the challenges of your system include needing the nitrogen tank and impaired visualization during the procedure. What are some strategies to address these issues?**

**BB:** Each system of ablation comes with its own potential challenges. However, the results physicians and patients see generally negate any challenges encountered. Our system does contain a liquid nitrogen tank that needs to be filled. We train each facility’s team how to do this safely. We are currently working on projects that would reduce the frequency of filling.

Loss of visualization when spraying does happen from time to time. Many physicians find it helpful to use a distal attachment cap and insufflation of CO_2_/air while spraying. Avoiding scope suction and water and maintaining proper distance when spraying helps decrease splash-back.

The challenges described can be mitigated with proper training and experience. Investing the time to train initially has been shown to be beneficial and provides more consistent treatment. Members of the STERIS truFreeze team train physicians and staff onsite to help get everyone off to a smooth start. Case support continues until the physician and staff feel comfortable with the process. It has been found that the average competency comes after about 5 procedures.^14^

Physicians often turn to truFreeze when all other treatment options have failed or have unpleasant side effects. Gastroenterologists can safely and effectively use cryotherapy with proper training.

**LL: What are the future plans for the cryoablation system? What things are you working on next?**

**BB:** Most of the research conducted on LNSC has been organic and physician led. When this happens, we get really excited about the technology because we know they are seeing results in practice, and they want to quantify it through clinical research. What’s next for us is to build on this research in GI and pulmonary using level 1 randomized trials that we hope will impact the standard-of-care guidelines and treatment approaches for many of the clinical applications we discussed in this interview. In parallel, you will see us innovate with cryogenics by expanding our cryotherapy offering with products that are easier to use and treat new areas of the body.

Thank you for this opportunity to share our experiences with LNSC.

## Editor’s closing remarks

Similar to RFA, the development and success of EC highlights the significance of embracing a collaborative approach and leveraging insights from diverse disciplines and industry in helping manage complex disease states. The role of industry and our partnership with them, focused on the development of novel therapeutic modalities for improved patient care, holds tremendous promise for addressing unmet clinical needs now and in the future. The integration of EC into clinical practice relies on collaboration between physicians across multiple disciplines to ensure that these advancements are effectively translated into improved patient care. Compared to RFA and other endoscopic ablation modalities currently in use, cryoablation holds more promise in treating cancers because of its greater depth of injury with potentially fewer adverse events. However, further studies as well as improvement in the technology are necessary to evaluate and optimize the role of EC in treating various benign and malignant GI conditions, including novel applications in evolution.

## Disclosure

The following authors disclosed financial relationships: L. S. Lee: Consultant for Boston Scientific, Fujifilm Medical, and Fractyl. B. Busby: Vice president and general manager of STERIS. V. Kaul: Consultant for STERIS.
